# Towards improved socio-economic assessments of ocean acidification’s impacts

**DOI:** 10.1007/s00227-012-2031-5

**Published:** 2012-08-21

**Authors:** Nathalie Hilmi, Denis Allemand, Sam Dupont, Alain Safa, Gunnar Haraldsson, Paulo A. L. D. Nunes, Chris Moore, Caroline Hattam, Stéphanie Reynaud, Jason M. Hall-Spencer, Maoz Fine, Carol Turley, Ross Jeffree, James Orr, Philip L. Munday, Sarah R. Cooley

**Affiliations:** 1 Centre Scientifique de Monaco, Avenue Saint-Martin, 98000 Monaco, Principality of Monaco; 2 IAEA EL, 4, Quai Antoine 1er, 98000 Monaco, Principality of Monaco; 3 LEA CSM-CNRS 647 ‘Biosensib’, Monaco, Principality of Monaco; 4 Department of Biological and Environmental Sciences, The Sven Lovén Centre for Marine Sciences, Kristineberg, University of Gothenburg, 45178 Fiskebäckskil, Sweden; 5 IPAG Lab, Nice, France; 6 Fisheries Policy Division, OECD, Paris, France; 7 The Mediterranean Science Commission, CIESM, Monaco, Principality of Monaco; 8 National Center for Environmental Economics, US Environmental Protection Agency, Washington, DC USA; 9 Plymouth Marine Laboratory, Prospect Place, The Hoe, Plymouth, PL1 3DH UK; 10 Plymouth University, Drake’s Circus, PL4 8AA UK; 11 Bar Ilan University, Ramat-Gan, Israel; 12 C3, Faculty of Science, School of the Environment, University of Technology, Sydney, NSW Australia; 13 Laboratoire Des Sciences Du Climat et de l’Environnement CEA-CNRS-UVSQ, Gif-sur-Yvette, France; 14 ARC Centre of Excellence for Coral Reef Studies, Townsville, QLD 4811 Australia; 15 School of Marine and Tropical Biology, James Cook University, Townsville, QLD 4811 Australia; 16 Woods Hole Oceanographic Institution, Woods Hole, MA USA

## Abstract

Ocean acidification is increasingly recognized as a component of global change that could have a wide range of impacts on marine organisms, the ecosystems they live in, and the goods and services they provide humankind. Assessment of these potential socio-economic impacts requires integrated efforts between biologists, chemists, oceanographers, economists and social scientists. But because ocean acidification is a new research area, significant knowledge gaps are preventing economists from estimating its welfare impacts. For instance, economic data on the impact of ocean acidification on significant markets such as fisheries, aquaculture and tourism are very limited (if not non-existent), and non-market valuation studies on this topic are not yet available. Our paper summarizes the current understanding of future OA impacts and sets out what further information is required for economists to assess socio-economic impacts of ocean acidification. Our aim is to provide clear directions for multidisciplinary collaborative research.

**Electronic supplementary material:**

The online version of this article (doi:10.1007/s00227-012-2031-5) contains supplementary material, which is available to authorized users.

## Introduction

The ocean reservoir of carbon is much greater than the terrestrial and atmospheric systems combined and provides an important net sink for carbon through exchanges of CO_2_ with the atmosphere. Over the past 200 years, atmospheric CO_2_ has increased from 280 ppm to a global average of nearly 390 ppm as a result of fossil fuel emissions, cement manufacture and land use changes. Carbon uptake by the ocean has slowed the atmospheric increase and its associated consequences for the Earth’s climate: without such uptake, it is estimated that atmospheric CO_2_ would now be around 450 ppm (Sabine et al. 2004; Le Quéré et al. 2009).

**Fig. 1:**
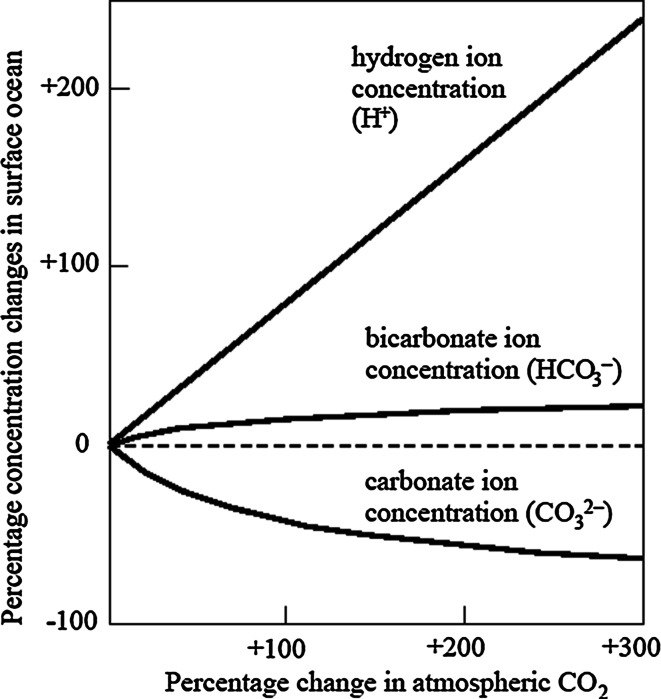
Percentage changes in average global surface ocean ion concentrations resulting from up to a fourfold change (300 % increase) in atmospheric carbon dioxide, compared with pre-industrial values. Values for atmospheric CO_2_ change from 280 to 1,120 ppm; bicarbonate ions from 1,770 to 2,120 μmol kg^−1^; carbonate ions from 225 to 81 μmol kg^−1^; and pH from 8.18 to 7.65 (where pH is defined as the negative decimal logarithm of the hydrogen ion activity, a linear relationship is assumed between activity and concentration, and a uniform and constant upper ocean temperature is assumed, of 18 °C). From Williamson and Turley (in press)

The increase in the rate of addition of CO_2_ to seawater by air-sea gas exchange due to increasing anthropogenic CO_2_ in the atmosphere is leading to an increase in hydrogen ion (H^+^) concentrations, and hence a fall in pH. Dissolved CO_2_, carbonic acid and bicarbonate are also increasing; however, the concentration of carbonate ions is decreasing as a result of a reaction between CO_2_ and carbonate, further increasing bicarbonate levels (Raven et al. 2005). The relative changes in bicarbonate, carbonate and hydrogen ion concentrations in the surface ocean arising from doubling, tripling and quadrupling of atmospheric CO_2_ (compared to pre-industrial values) are shown in Fig. 1. Uptake of this additional CO_2_ has already increased the average acidity of the global ocean by 30 % (decreasing pH from 8.2 to 8.1) since the beginning of the Industrial Revolution, and the increase in acidity is expected to increase threefold (yielding a decrease in pH to 7.8) by the end of this century if CO_2_ emissions continue at current rates. Therefore, absorption of CO_2_ by the oceans at a rate of 25 million tons of CO_2_ per day contributes to the mitigation of global warming, but at a cost to ocean carbonate chemistry (Fig. 1). Ocean acidification (OA) is the term used to describe these changes in ocean chemistry.

It is anticipated that OA could have dramatic consequences this century, potentially including extinction of keystone marine species (Dupont et al. 2008). Organisms producing a carbonate shell or skeleton have been the primary research focus to date, as calcification partly depends on carbonate ion concentration. Evidence is increasing that organisms respond to OA to variable degrees (Ries et al. 2009; Hendriks et al. 2010; Fabricius et al. 2011) and that sensitivities differ between species, with some species showing negative responses while others show no or positive responses (Dupont et al. 2010; Ferrari et al. 2011a). Direct consequences of decreasing seawater pH, such as changes in growth rates, are mediated through changes in acid–base status or shifts in energy budget. These effects also depend on species-specific capacities to compensate. Additional physiological disturbances have been reported, including behavioural changes, reductions in fertilization and reproductive success through development effects to eggs, larvae and juveniles (Gattuso and Hansson 2011a). Impacts from OA can be modulated and exacerbated when combined with other environmental parameters such as food availability, temperature increases or hypoxia (Pörtner and Farrell 2008; Thomsen et al. 2010; Gruber 2011). Projected future changes at the ecosystem level could include relative shifts in fitness and competitiveness, changes in species interactions and biogeography, and ecosystem restructuring due to synergistic effects with temperature and changes in species composition and biodiversity. Marine organisms also may be indirectly at risk due to OA’s effects on key components of food webs, for example, on phytoplankton quality (Rossoll et al. 2012) or specific zooplankton such as pteropods that are essential in the diets of salmon and whales (Orr et al. 2005). There has been a growing awareness that many of the goods and services provided by the ocean may be at risk from increasing OA, and for this reason, there could be far-reaching socio-economic consequences (Turley and Boot 2010, 2011; see Table 1 in supplementary data).

In 2010, an international workshop in Monaco on the socio-economic impacts of OA, organized jointly by the Centre Scientifique de Monaco and the International Atomic Energy Agency, brought economists and scientists together for the first time. The aim was to start building the methodology for integrating knowledge from these different disciplines to better study the socio-economic impact of OA. Here we summarize some of the findings from this workshop. We review the rationale for a multidisciplinary approach between natural and human sciences; the data required for it; the current knowledge gaps and limitations; and major uncertainties concerning future impacts on species, ecosystems and the goods and services they provide.

## Motivation for a multidisciplinary approach

When designing efficient policies to address CO_2_-linked environmental change, issues spanning multiple temporal and spatial scales must be taken into consideration. Reductions in CO_2_ emissions large enough to avoid the worst consequences of OA and climate change will require global economy-wide emissions policies and international agreements (Turley and Gattuso 2012). These are likely to be costly and necessitate trade-offs within other sectors of the economy. Higher energy prices, for example, may slow economic growth, meaning a lower standard of living for many people in the near term. So when policy makers consider emissions policies, they need to weigh the benefits that will be realized far into the future with the social costs that will begin to accrue almost immediately. Whether emission reductions are achieved through economic instruments, such as a carbon tax or cap and trade programme, or through technology mandates, those policies are likely to carry with them significant costs (e.g. OECD 2008; Paltsev et al. 2009; Tol 2010). At the same time, effects of OA and climate change will likely play out on individual marine resources, disproportionately affecting certain groups of people with high dependence on affected ecosystems.

**Fig. 2:**
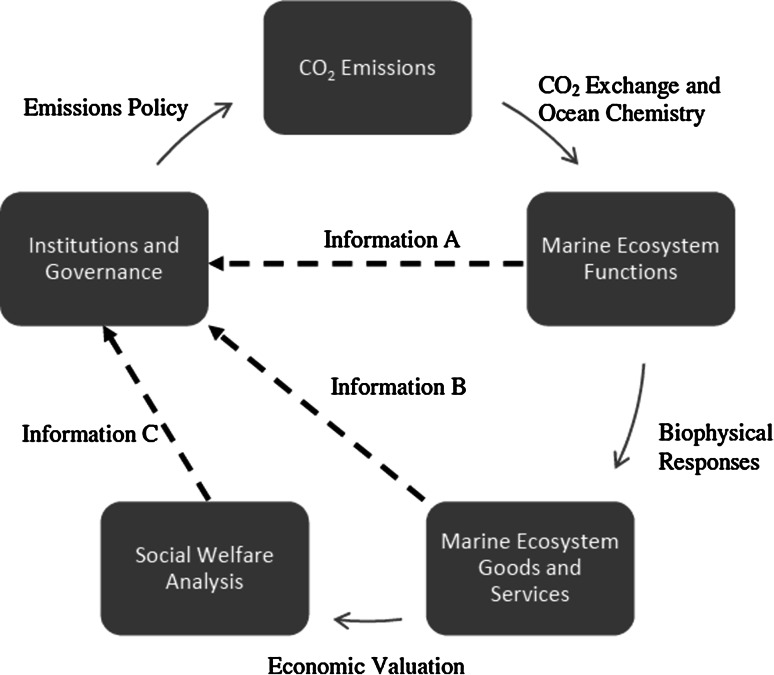
CO_2_ impact pathway and flow of information

Figure 2 is a conceptual schematic showing how policy decisions, ecological impacts and social welfare are related in the context of CO_2_-emissions-linked ocean changes such as ocean acidification. The solid arrows represent pathways through which CO_2_ emissions impact marine ecosystems and the human systems that rely on them. The dashed lines are flows of information to policy makers whose charge is to maximize social welfare. Policy makers can base decisions on observations of the natural system (information A and B) or on economic analyses that express costs and benefits of emissions policy in monetary terms (information C). The latter provides the most comprehensive set of information but also requires knowledge embodied in information A and B. This figure also demonstrates the relevance of economic valuation and shows how knowledge of welfare impacts can be used directly by the policy maker, or the natural resource manager, to support policy action since the absence of action will often be associated with high welfare damages (as quantified by the information flow C)—see Chiabai et al. (2011) for an example of an economic valuation exercise.

## What data do economists need for research on OA?

### Relating carbon emissions policy to changes in seawater chemistry

The first step towards understanding the socio-economic impacts of OA and what they imply for emissions policy choices is better information on the atmosphere–ocean carbon flux, particularly in estuarine and coastal environments. Atmospheric CO_2_ concentrations and radiative forcings from all greenhouse gas emissions can be modelled under a variety of socio-economic scenarios providing forecasts of *p*CO_2_ and temperature. Given a handful of other physical and chemical seawater variables, the resulting exchange of CO_2_ between the atmosphere and the open ocean can be modelled with a reasonable amount of confidence (e.g. Orr et al. 2005).

The majority of human activity, including recreation, commercial and subsistence fishing, and aquaculture occur in estuaries and coastal areas where the carbon dynamics are not well understood. Seasonal patterns in CO_2_ under- and oversaturation have been measured in coastal zones and have been linked to changing thermal and biological conditions (Borges and Frankignoulle 2003). A number of additional influences on seawater chemistry are also present, such as freshwater inputs that may be loaded with nutrients and contaminants, or acid precipitation in coastal seas (Doney et al. 2007). It has been suggested that eutrophication may be a stronger driver of carbonate chemistry change in coastal wasters than the uptake of CO_2_ by seawater (Borges and Gypens 2010), but this has not been determined for all environments. More reliable forecasts of conditions in these areas are critical to understanding the potential socio-economic impacts from OA for the purposes of economic analyses.

### Biophysical responses to OA

To provide policy makers with the most immediately relevant information, economists would benefit from some changes in the approach that has been taken in research observing how marine organisms respond to changing seawater chemistry. Understanding fitness-related impacts on economically important species is the simplest way to link OA to changes in social welfare. If credible research shows that OA can have unambiguous effects on species that humans culture, harvest and/or consume (e.g. molluscs, crustaceans, fish), this information will be directly useful for economists and policy makers. To date, relatively few species, most of them without any economic importance, have been studied while the few data available indicate that there is a diverse range of interspecific responses to OA (Ries et al. 2009; Dupont et al. 2010; Ferrari et al. 2011a), making generalization difficult.

However, while it is important to investigate the sensitivity of economically relevant species, it is also important to study taxa with no commercial value but which provide critical habitat and occupy important trophic levels within marine food webs (Guinotte and Fabry 2008). Direct economic effects also result from OA-induced changes in important marine habitats, such as coral reefs (both deep and shallow) and other biologically mediated environments (e.g. maerl and mussel beds). Reefs contribute to a number of ecosystem services such as leisure and recreation, and coastal protection, as well as the provision of shelter and food for a multitude of other marine species (Coen et al. 2007; Moberg and Folke 1999). For coastal developing countries that are highly dependent on tourism as a source of foreign income, loss or degradation of coral reefs, for example, could have potentially larger effects than losses in mollusc harvests.

In addition to shifting the focus of studies towards economically important species, economists would also benefit from observations of economically relevant measures of the biophysical impacts. For example, people buy molluscs and crustaceans because of what they find inside the shell, not for the shell itself. Studies that examine the effect of OA on the edible or exploitable (e.g. pearl in oysters) portions of affected species are more relevant to economic analyses than those that report changes in other processes such as calcification. These parameters should also be considered in the light of long-term sustainable production for aquaculture and harvest in wild populations. For economists to benefit from research findings in other scientific disciplines, these must be clearly presented in a way that underlines their relevance to socio-economic studies.

### Informing emissions policy with economic analysis

Once the links between CO_2_ emissions and marine ecosystem impacts have been established, there are several ways that data can inform policy. Some ecosystem impacts can be expressed in monetary terms, following the “Economic Valuation” arrow in Fig. 2 to Social Welfare Analysis. This is the ideal situation because, if properly carried out, social welfare analysis reveals how resources, whether they are marine ecosystem services, fossil fuels, fish stocks, to name but some, can be employed to benefit society the most. However, not all impacts can be expressed in monetary terms. Impacts such as these that can be quantified in terms other than economic value or only expressed qualitatively inform policy and governance via the “Information B” arrow in Fig. 2. Once scientists are able to link increases in atmospheric CO_2_ to changes in the availability of goods and services provided by the ecosystem, economic modellers can begin to quantify those impacts and present policy makers with the information they need to craft economically efficient carbon policy.

Some ecosystem impacts for which reliable valuation methods exist can be evaluated in terms of market impacts, that is, how environmental degradation affects markets through prices and costs. These can be measured by observing how people react to increasing prices and decreasing quantity and quality of commodities that they purchase. Such impacts can be analysed with the standard tools of economic theory (Varian 1992; Mas-Colell et al. 1995). For example, Dey (2000) analyses the demand for fish in Bangladesh and examines the welfare impacts of technological and policy changes in the fisheries and aquaculture sectors. Studying how consumption of marine resources responds to price and income changes can provide insight on the likely welfare impacts. However, most of the studies conducted to date were not performed with OA impacts in mind and so their utility in this application is limited. Economic theoretical models map the effect of changes in supply to changes in economic values, taking into consideration market responses. Such models are based on equilibrium analysis and can be extended to include uncertainties, both regarding the underlying data as well as the model specification itself (robustness). Initial studies that explore potential market changes are emerging (Cooley and Doney 2009; Narita et al. 2011), but are still limited in scope. They focus solely on mollusc production and make considerable extrapolation from short-term single-species experiments and assumptions about the relationship between calcification in molluscs and their growth and productivity.

Nonmarket impacts, on the other hand, reflect the effects of environmental degradation on ecosystem goods and services that are not traded in traditional markets and do not have explicit prices attached to them (for a review on the theory and practice of ecosystem services and their assessment see Daily and Matson 2008). Some of the non-market goods and services that are likely to be affected by OA are cultural services such as recreational fishing, diving, contribution to arts and religion, and so on (see de Groot et al. 2010 for an expanded list), but also regulatory services such as shoreline protection and coastal stabilization (Cooley et al. 2009), as well as the biological remediation of waste and the ability of the marine environment to sequester carbon. To determine a change in the provision of non-market services, economists require data on how much money people are willing to pay to preserve them or replace them. There are a variety of methods that economists use to estimate non-market values (for a review see Champ et al. 2003). To date, few studies have attempted to value such marine non-market goods and services in the context of OA.

### Need for other social science data and methods

More reliable data on the production and consumption of market and non-market goods affected by OA would improve economists’ ability to quantify the social welfare impacts. These data would include the quantities and prices of affected marine species and how the costs of production are likely to change in the future. For example, OA is known to make maintenance more energetically expensive with organisms making trade-offs between processes such as calcification and growth. Within aquaculture, if growth is to be maintained, feeding costs are therefore likely to increase to compensate for the increased energy demand. Hatcheries that can maintain water pH at higher levels may also be required to ensure a continued supply of spat for aquaculture (Kite-Powell 2009). The costs of maintaining such water quality are not well established. Furthermore, it can be assumed that the costs of coastal protection may increase if such coastal protection is dependent upon coral reefs, or other biologically mediated environments comprised of organisms that may be susceptible to OA.

There is also a need to understand how society may respond and adapt to potential changes resulting from OA. Modelling efforts need to explore two-way relationships between the marine environment and humans. The typical approach to modelling examines how human activities drive change in the marine environment and then identifies the consequences for society, sometimes through linked bioeconomic models. Few go on to explore how human activities change as a result of marine environmental change (Barange et al. 2010). Novel approaches, however, are available that could be applied to OA impacts, such as end-to-end models. For example, Merino et al. (2010) developed a global small pelagic fish model to investigate the potential impact of different climate change scenarios on fishmeal supply using changes in primary production as a driver of change. They found that the sustainability of the small pelagic fisheries depends more on societies’ response to climate change than on the magnitude of climate change impacts themselves. The same could be true for OA. Many end-to-end models, however, are limited by their focus on single drivers of change (e.g. temperature change), and greater effort is required to incorporate multiple drivers and cumulative effects (e.g. Fulton et al. 2011).

Recognizing how people may adapt and what coping strategies they use to do so may be particularly important for understanding the impacts of OA on society. For example, Cooley et al. (2012) indicate that a number of developing countries, especially Small Island Developing States, may be particularly vulnerable to losses in mollusc harvests for nutritional and/or economic reasons. At the same time, many of these countries may also be vulnerable to climate change impacts on fisheries (Allison et al. 2009; Bell et al. 2011). As these nations experience losses, they will undertake adaptive strategies that will address the array of multiple stressors acting on their resources. The end-to-end models currently in use will not necessarily realistically capture those natural human responses.

### Research priorities for estimating socio-economic impacts of OA

Economists need specific types of high quality empirical data on how OA will affect the ecological production functions for the most important goods and services that are likely to be affected. To build more reliable economic models using this information, a better understanding of carbon dynamics in estuaries and near-shore areas (information A in Fig. 2) is first needed. Information on ecologically, economically and socially important species and habitats and economically relevant traits is needed. There is also a need to understand how society may itself adapt to changes resulting from OA. While the last request falls outside the purview of natural scientists, the flow of information represented in Fig. 2 would be incomplete without reliable data on the consumption and production of both market and non-market goods and services that will be affected by changing seawater chemistry. Furthermore, it is necessary to have reliable estimates on the time span of different scenarios. Time plays a crucial role in economic analysis when comparing different scenarios and policies where both costs and benefits occur at different points in time (Gollier 2002). What data are needed also depends on the scale of the economic analysis, that is, whether it is applied to a local community or a regional, national or international level. Conversely, data availability can influence the scale of the economic analysis possible. The final dataset needed concerns estimation of uncertainties regarding different scenarios. Having some measures of uncertainty makes it possible to estimate the risk associated with different scenarios (for a classic discussion on risk and uncertainty see Knight 1921; for a more recent text, see Mas-Colell et al. 1995).

## What biological data are currently available to economists?

In this section, we briefly review what is known, data gaps and highlight limitations of the methods used and suggest future research priorities. It is not the goal of the present paper to review biological effects; however, some general trends are presented. For a comprehensive review see Gattuso and Hansson (2011a).

### Short summary of biological impacts

The biological impacts of OA on marine organisms are only beginning to be understood. Impacts of OA may be either direct, through alteration of different physiological processes (survival, reproduction and development, growth, metabolism, thermal tolerance, immune response, behavioural responses, etc.), or indirect, through ecological interactions (predator–prey abundance, nutrient recycling or habitat changes). Indirect impacts are, however, harder to predict because of the complexity of ecological interactions. Because of the initial hypothesis that OA will primarily alter calcification through changing seawater carbonate concentration and saturation state of calcium carbonate, the first experimental work focused on calcifying organisms (Fabry 2008). It appears now that many other biological functions are sensitive to OA extending to almost all forms of life, from fish to bacteria (Gattuso and Hansson 2011a). The impact of OA can be severe and can potentially lead to species extinction (e.g. Dupont et al. 2008). However, the effect on a single organism is not always negative; phytoplankton photosynthesis is generally enhanced by OA (Riebesell and Tortell 2011), and some seagrass species even thrive in low-pH environments (Hall-Spencer and Rodolfo-Metalpa 2008). For coral, most studies have shown a decrease in growth, but this effect seems also to depend on temperature (Reynaud et al. 2003) and species (Fabricius et al. 2011). Calcifying organisms such as crustaceans, echinoderms and molluscs show a range of responses to OA from negative (50 % of all tested species) to zero or even positive effects (e.g. Dupont et al. 2010). In conclusion, we have enough evidence demonstrating that OA can impact marine organisms, including causing species extinction, and we can forecast likely consequences for marine ecosystems. However, these impacts are highly species- and even population-specific.

Information from single-species experiments is often used to make predictions of the impact at the ecosystem level (e.g. Troedsson et al. this issue). However, work on naturally rich CO_2_ habitats illustrates the risk of upscaling from single-species experiments without considering the complexity of ecological interactions. Single-species short-term perturbation experiments do not integrate the effects of climate change across entire systems (Russell et al. 2011). For example, a negative impact on a given parameter observed in the laboratory may not translate to a negative impact on the species fitness, and ecological interactions can modulate a species-level response. The importance of ecological interactions is illustrated by the work performed on areas that are naturally enriched with CO_2_ such as CO_2_ vents (Hall-Spencer et al. 2008; Fabricius et al. 2011; Rodolfo-Metalpa et al. 2011), hydrothermal vents (Couto et al. 2010; Vizzini et al. 2010; Bianchi et al. 2011) and upwelling areas (Thomsen et al. 2010). Vents off Italy and off Papua New Guinea have been shown to enrich seawater CO_2_ levels and alter calcification, recruitment, growth, survival and species interactions in the acidified waters (Hall-Spencer et al. 2008; Fabricius et al. 2011). Many species of microalgae, macroalgae, seagrass, foraminifers, corals, polychaetes, crustaceans, molluscs and bryozoans are remarkably tolerant of long-term exposures to high and variable carbon dioxide levels at the vents (Kroeker et al. 2011; Johnson et al. 2012). However, a fall in mean pH from 8.1 to 7.8 can have detrimental effects on the recruitment of benthic organisms from the planktonic forms (Cigliano et al. 2010). Adult populations show dramatic reductions in biodiversity along gradients of increasing CO_2_ in coastal Mediterranean systems and in tropical coral reef systems, with around 30 % fewer species in adult populations at a mean pH of 7.8 than in adjacent areas at a mean pH of 8.1 (Fabricius et al. 2011; Kroeker et al. 2011). Important groups, such as coralline algae, calcified foraminifers and sea urchins are common outside the vent systems but absent from areas with mean pH ≤ 7.8, probably due in part to widely variable carbon dioxide levels. On the other hand, mussels and other calcifiers predicted to be negatively impacted by OA based on perturbation experiments can dominate CO_2_-enriched environments where food sources are abundant (Thomsen et al. 2010).

### Methods used to study biological impacts: importance and limitations

Historically, perturbation experiments were the first method used to study the effect of pH on marine organisms. pH was recognized as early as the beginning of the nineteenth century as an important parameter affecting both land and marine animals (for an historical perspective concerning marine animals, see Gattuso and Hansson 2011b). Perturbation experiments have been mainly performed in laboratory conditions by incubating organisms of a given species at different stages of their development (adult, egg, larvae), or, more rarely, assemblages of several species (mesocosms). The length of the incubation period has been generally short (from 1 h to 1 year, but most of them last from a few days to a few weeks). A vast majority of these experiments only considered OA without any synergy with other stressors or including natural variability. At the end of the incubation period, experimental organisms were compared to organisms maintained in control conditions using present-day CO_2_ conditions. Several biological parameters were surveyed: survival, growth, calcification, respiration, photosynthesis, metabolic activity, morphology, gene expression, etc. A small number of perturbation experiments have been performed directly in large mesocosms in the field (e.g. Bellerby et al. 2008; Schulz et al. 2011). More recently, the discovery of naturally high CO_2_ environments allowed in situ observations. However, these sites do not fully mimic OA conditions as they are generally small in scale and are constantly fed with individuals and larvae from a non-enriched habitat.

Even with the number of studies increasing at an exponential rate (see Fig 1.2 in Gattuso and Hansson 2011a), comparing these data is extremely difficult owing to the use of different experimental protocols (conditions of incubation: duration, methods of acidification, environmental parameters) for different species and different developmental stages. However, the publication of the EPOCA best practices guide for OA research (Dickson et al. 2007) is a step forward in the development of best practices and standardization of experimental methods.

## Recommendations for future data collection to inform socio-economic OA studies

### Ocean chemistry

#### Collect observational data

Ongoing changes in surface pH, *p*CO_2_ and CaCO_3_ saturation states are measureable at time-series stations, which tell us about trends as well as daily, seasonal, interannual and decadal variability. Measured surface ocean trends agree with what is expected from the measured atmospheric increase in carbon dioxide, assuming air-sea CO_2_ equilibrium. Yet the high-precision chemical measurements that are needed over decadal time scales are only available at a very limited number of stations confined mostly to the northern subtropics of the open ocean. It is crucial to extend this network to other key regions, particularly the high-latitudes, marginal seas and coastal areas, which are generally more variable, more vulnerable and of greater economic importance.

There is also a critical need for well-developed spatial and temporal models that give accurate present-day and future estimates of aragonite and calcite saturation states in the coastal zones. The shallow continental shelves are some of the most biologically productive areas in the sea and are home to the majority of the world’s fisheries, but accurate saturation state data do not currently exist for most coastal regions (Guinotte and Fabry 2008). Future work should focus more on measured variability, including oscillations on short-term biologically relevant to decadal time scales, to characterize driving mechanisms and to better evaluate models that are used to make future projections (Hofmann et al. 2011; Shaw et al. 2012; Doney et al. 2009). Time-series stations are complemented by large-scale sections in the open ocean that extend well beyond the subtropics. Some of these sections have been reoccupied to assess trends; their subsequent resampling will need to be repeated roughly every 10 years to adequately follow increasing OA.

#### Develop observational and predictive technology

The greatest opportunity for a leap in understanding variation in ocean chemistry would come from further developing new sensors and assuring that they are deployed widely. New in situ high-precision sensors are being developed for pH and related biogeochemical variables, including oxygen, nitrate, partial pressure of CO_2_ (*p*CO_2_), and particulate inorganic carbon. More effort is needed to refine precision and stability of these sensors, develop others especially for key carbonate system variables (e.g. dissolved inorganic carbon and alkalinity), and deploy them not only on moorings but in a new networks of gliders and floats. The ARGO array, now with more than 3,000 operational profiling floats spread randomly throughout the ocean, each measuring temperature and salinity in the upper 2 km of the ocean every 2 days, has revolutionized our ability to characterize the ocean’s circulation and warming. Likewise, equipping many ARGO floats with multiple biogeochemical sensors would revolutionize our ability to characterize the ocean carbon cycle and quantify how it is being affected by OA.

Although essential for assessing the current state of the ocean, measurements are also helpful for improving model projections. Routinely, measurements are used to evaluate models and adjust model parameters. In addition, if data coverage is sufficient to characterize the modern mean state of the real ocean, the bias in the model’s modern mean state (model minus data) can be removed, improving the future projection. This approach substantially improves model agreement of projected chemical changes in the open ocean due to OA (Orr et al. 2005), but it only lends itself to regions with extensive data coverage. Also, the more recent the observed mean state, the more reliable the future projection. Thus, the best projections will require frequent updates of extensive global data sets, a task that may well require an international observing system. If it existed, that observing system would be exploited as part of an international model-data comparison. Together, these international efforts would strengthen links between modellers and data experts, thereby improving projections of acidification in the context of other changes (e.g. warming, stratification, deoxygenation) and providing a forum to share advances, for example, to account for more complex environments or processes.

#### Determine biological relevance of chemical changes

Once enhanced data collection establishes the natural regional limits of temporal and spatial pH and saturation state variability, the next step is to identify what constitutes biologically relevant change. Many economically important coastal species are accustomed to wide pH variations and some even spend time in environments undersaturated with respect to calcite and aragonite (Green et al. 2009). Current hypotheses suggest that different species have different tolerances for time spent at lowered pH. Maps can be made of model-projected pH and saturation state (Feely et al. 2009) that depend on carbon dioxide emissions scenarios. But it is uncertain whether species will respond to these chemical changes or whether they will be sensitive to relative changes (the magnitude of change from present conditions) (Feely et al. 2009). Therefore, it is not possible yet to develop maps indicating economically relevant species’ susceptibility to OA based on mechanistic responses. To do this, more information is needed from biological studies in laboratories and in the wild that relate the success of economically relevant biota to present chemical conditions and change in them, in addition to enhanced chemical datasets. This will also be complicated by the need to integrate the impact of chemical changes on other species connected (e.g. food, competitor, predators) to the species of interest.

### Quantify marine biology

#### Perform long-term and multigenerational experiments

To date, the majority of published OA research involves short-term experiments (days to weeks) that acutely stress organisms using predicted future ocean conditions. Although such experiments are useful for studying physiological processes and identifying major biological effects, they may not provide a true assessment of the longer-term impacts of OA. First, some effects of exposure to acidified conditions may take months to appear. For example, increased costs associated with high CO_2_ and low pH may only become evident in whole-organism traits such as growth and survival after several months of continuous exposure (e.g. Langenbuch and Pörtner 2004; Shirayama and Thornton 2005; Kurihara et al. 2008). Alternatively, organisms might acclimate to acidification over time. For example, the crab *Necora puber* used bicarbonate ions acquired from shell dissolution to regulate extracellular pH during a short-term (14 days) exposure to elevated CO_2_ (Spicer et al. 2007); however, bicarbonate was acquired from the surrounding seawater to regulate pH after longer-term exposure (30 days) (Small et al. 2010). One of the key questions is to determine what is the relevant time of exposure. In a study on the green sea urchin *Strongylocentrotus droebachiensis,* Dupont et al. (2012) show that female fecundity was decreased 4.5-fold when acclimated to elevated *p*CO_2_ for 4 months during reproductive conditioning, while no difference was observed in females acclimated for 16 months. Therefore, short-term experiments can both over- and underestimate the impact of OA.

The rate of change to an environmental stressor (rapid/abrupt *vs* gradual changes) can also modulate selection pressure and result in various strategies for adaptation. The contrast between so-called hard and soft selection is a key concept in evolutionary biology, but has received little attention in the marine environment in general and OA research in particular. Most perturbation experiments published so far used abrupt changes in pH. However, previous work on the *p*CO_2_ changes on terrestrial ecosystems have shown that abrupt changes can lead to an overestimate of the real impact on fitness (Klironomos et al. 2005). More realistic experimental design is needed to fully mimic near-future OA.

Experiments should also include subsequent life history stages (e.g. gametes–embryos–larvae–juveniles–adults) that may be critical in understanding the effects of environmental stressors on marine species (Marsahll and Morgan 2011). Carry-over effects between life stages can significantly affect the outcome of environmental stress in subsequent life stages. For example, pre-exposure of adult sea urchins to elevated *p*CO_2_ had a negative impact on subsequent larval settlement success. Five to nine times fewer offspring reached the juvenile stage in cultures using gametes collected from adults previously acclimated to high *p*CO_2_ for 4 months. Furthermore, *p*CO_2_ had no direct negative impact on juvenile survival except when both larvae and juveniles were raised in elevated *p*CO_2_. These negative effects on settlement success and juvenile survival can be attributed to carry-over effects from adults to larvae and from larvae to juveniles (Dupont et al. 2012). On the other hand, positive carry-over effects are also possible. Parker et al. (2012) found larvae from parents exposed to elevated *p*CO_2_ during reproductive conditioning were larger, developed faster, had similar survival to current-day controls and were less impacted by exposure to elevated *p*CO_2_. Furthermore, recent experiments with reef fish have demonstrated that metabolic decrements caused by rising water temperature, which have severe effects on growth and development, can be fully compensated in future generations when parents are reared their entire life under future climate change scenarios (Donelson et al. 2012). A greater focus on long-term and multi–life stages experiments, especially for economically important species, is required to properly assess the impacts and cost of OA on marine ecosystems.

#### Explore synergy between stressors

Another pitfall concerns potential cross-effects between elevated CO_2_ and other relevant environmental parameters (e.g. temperature, salinity, oxygen concentration, etc. but also parameters co-varying with pH such as toxic compounds or heavy metals). Several studies demonstrate indeed a synergy between parameters, which are expected to change in the near future (e.g. for synergy between pH and temperature, Reynaud et al. 2003; Metzger et al. 2007). The combined action of acidification and other stressors is likely to worsen impacts that would be expected from acidification alone. Nutritional status of organisms is also rarely taken into account; nevertheless, it has been shown that it can greatly impact the final results (Holcomb et al. 2010; Melzner et al. 2011). Future OA will not occur in isolation from other types of impacts on the marine environment. Land-based point and non-point sources of pollutants are already problematic for the economic management of aquaculture and wild fisheries, particularly for developing countries that have higher economic dependencies on marine products. The co-occurrence of OA with land-derived contaminants may be detrimental to economic production functions for seafood via the following interactions: direct additive or synergistic effects on reproductive success; developmental and growth rates; enhanced accumulation of contaminants in edible tissues of contaminants; or by a direct effect of OA on bioavailability and toxicity of heavy metals, as pH may affect their solubility, adsorption and speciation (Millero et al. 2009; Pascal et al. 2010). OA-mediated elevations in body burdens of contaminants in seafoods can lead to enhanced exposure of their human consumers close to the source as well as restrictions in their regional and national trade and export. This can be detrimental to livelihoods if such enhanced contaminant levels exceed national and internationally accepted levels, as incorporated within *Codex Alimentarius*. Several recent experimental studies have reported on the possible interactions between projected future OA and co-contaminant accumulation and biokinetics in marine biota, including some significant categories of seafood of increasing importance, such as cephalopods. For example, it was shown that pH had an effect on the metal adsorption and protective properties of the eggshell and enhanced CO_2_ modified the metabolism of cuttlefish embryo and paralarvae. Both these factors caused changes to the accumulation of metals in the tissues of squid *L. vulgaris* (Lacoue-Labarthe et al. 2009, 2011).

Another example is the complex interaction between OA, immunity and diseases. Diseases and infections are a major problem for aquaculture and immunity can play an important role in stable maintenance of natural populations and sustainable aquaculture. Impact of OA on organism health status is still poorly understood, but of increasing concern because near-future synergy between different environmental changes (e.g. global warming, ocean acidification, increases of catastrophic meteorological events) can affect species immune response, population dynamics and the distribution of pathogens (Dupont and Thorndyke 2012).

#### Understand adaptation potential

A major obstacle to predicting the economic costs of OA on marine ecosystems is that almost nothing is known about the potential for marine species to adapt to rising CO_2_ levels and changing ocean chemistry over coming decades. While some scientists suggest that organisms have shown no signs of biological adaptation to OA in the past (see for example Veron 2008), new studies are demonstrating that some species may have enough phenotypic and genetic variation to cope with OA. For example, the sea urchin species *Strongylocentrotus franciscanus* has vastly greater levels of phenotypic and genetic variation for larval size in future CO_2_ conditions compared with the mussel species *Mytilus trossulus* (Sunday et al. 2011). Variation in the response of individuals to elevated CO_2_ and low pH is observed in many experiments (e.g. Dupont and Thorndyke 2009; Munday et al. 2010; Parker et al. 2011; Sunday et al. 2011), with some individuals in acidified conditions exhibiting similar performance to individuals in control conditions. In some cases, it is clear that this variation is heritable (Parker et al. 2011, 2012; Sunday et al. 2011), indicating that populations could develop increased tolerance to acidified conditions through time. Information on genetic and phenotypic variation, and key demographic parameters, may lend valuable insight into relative evolutionary potentials across a large number of species and is of high priority for future research.

#### Determine community and ecosystem responses

Although there is increasing evidence for impacts of OA on individual species and life history stages, a major challenge is predicting the community and ecosystem effects of OA. Existing results indicate that community and ecosystem responses cannot be adequately predicted by simply scaling up from studies of physiological processes or from studies of single species (but see Troedsson et al. in press). Individual responses may be very different in the presence of competitors and predators, and interacting effects of many species may be difficult to predict even with a reasonable knowledge of how individual species respond to OA in isolation. For example, Ferrari et al. (2011b) found that when juvenile fish and their larger predators were placed together in a mesocosm, the mortality rate of the juveniles was higher if both the predators and prey had been exposed to elevated CO_2_. This result confirmed predictions from previous research of behavioural effects of elevated CO_2_ on juvenile fish (Dixson et al. 2010; Munday et al. 2010) and their predators (Cripps et al. 2011). However, Ferrari et al. (2011b) also found that the prey species preferred under control conditions were not the same ones preferred under high CO_2_. This result obviously has important implications for understanding the effects of elevated CO_2_ on the community structure of reef fish assemblages, but could not have been predicted from the studies of predators and prey in isolation. Similarly, decades of physiological studies on acid–base regulation in fishes did not predict that their behaviour would be dramatically altered under high CO_2_, despite the behavioural changes that were caused by changes in the concentrations of acid–base relevant ions (Nilsson et al. 2012). Well-designed experiments that test the effect of OA on species interactions and key ecological processes (rather than just impacts on species) will be vital in attempts to scale up to higher levels of community and ecosystem complexity that are needed for economic assessments.

Ecosystem models are another useful tool for exploring community and ecosystem effects of OA (Fulton et al. 2011) that can be parameterized with existing and emerging knowledge and sensitivity-tested to identify the knowledge gaps that are most productive to fill. Naturally high-CO_2_ environments can also provide important information on how communities may change in the future (Hall-Spencer et al. 2008; Fabricius et al. 2011) to inform ecosystem models, although it must be recognized that their scale is small compared with the current-day scale of biological connectivity and the future scale of OA. Because these sites are constantly fed with individuals and larvae from non-CO_2_-enriched habitats, the potential for selection is weakened and thus potentially overestimates the impact of *p*CO_2_. All approaches should be encouraged and opportunities to incorporate different approaches (e.g. combining community experiments and models or natural analogues and experiments) should be identified.

It is important to note that scaling up ecosystem data and models to consider socio-economic outcomes has its challenges: both OA’s effects on marine ecosystems and its socio-economic implications strongly depend on local and regional contexts (CIESM 2008). Even if marine conditions in one site are analogous to those elsewhere, each region will have different governance, economic activities, and stakeholders, all of which will interact in varying ways with OA-affected ecosystems.

## Identify potential solutions

Scientists may provide information on impacts, but they may also explore potential ways to limit or mitigate negative impacts of OA. For example, it could be possible to explore a species genetic diversity to isolate “OA-proof” tolerant strains. There is a growing body of evidence showing that sensitivity to OA varies within species, some genotypes being more resilient than others. Isolation of resilient strains of species with ecological and/or economic importance may improve future aquaculture and/or rescue threatened ecosystems. This approach was investigated by Parker et al. (2011) showing that fast growing strains of oysters were potentially more resilient to OA. Another option is to determine key areas for conservation efforts. Even if the cause of OA is global and cannot really be controlled at the local level, reducing local stresses acting on ecosystems can improve their resilience to all stressors, including OA (Hughes et al. 2010). Key areas for conservation efforts should be selected on both biological/ecological and economic bases. Even though they do not directly mitigate OA, natural coastal carbon sinks (kelp forests, mangroves, seagrass beds, salt marshes) are critical areas to include in future carbon management discussions and strategies. Furthermore, bearing in mind the complexity and the diversity of OA’s effects on a wide range of economic activities, proposed areas for conservation should be characterized by flexible and accommodating governance structures where each of the involved stakeholder groups can discuss and agree on policy/management instruments and propose mutually agreeable conservation efforts. With some exceptions (see Samonte et al. 2012), few studies exist in this domain. This gap is currently being addressed *inter alia* by the ongoing EU project *The European Mediterranean Sea Acidification* in a changing climate (MedSea), which is funded by the European Commission under Framework Program 7 involving 16 institutions from 10 countries and which explicitly addresses the economic valuation of OA impacts along with proposing adaptation tools and policies to limit the socio-economic impact of acidification on the Mediterranean area (MedSea 2012).

## Conclusions

It thus appears necessary to undertake a quantitative assessment of the likely biological, social and economic (or “bio-socio-economic”) risks and vulnerabilities that may result from OA. This assessment would be supported by the following information for each country: (a) percentage of GNP that is reliant on coastal production of seafoods, aquaculture production and coastal tourism that depend on OA sensitive ecosystems; (b) composition of seafood relative to its likely vulnerability to OA; (c) projected increases in coastal zone-dependent populations to 2100; (d) proportions of coastal populations living close to the poverty level, where the detrimental effects of OA would be more acutely felt; and (e) the vulnerability and sensitivity of these coastal populations to environmental change and an assessment of their capacity to adapt. With this information, integrated assessment models can be developed that will demonstrate the social welfare impacts of different CO_2_ emissions policies on specific regions of the world. Such a model would trace the impact pathway of CO_2_ emissions through the changes in biophysical processes to market and non-market impacts on social welfare. They have been developed for the impact pathway of CO_2_ on climate change (Tol 2002; Nordhaus 1994; Hope et al. 1993) and are being used by the USA, UK and European Union to craft emissions policy.

It is essential that biologists and economists work together to enable policy makers to base their policies on objective and precise elements: “we need you; you need us” should be the slogan unifying our two communities. Even if it is now sure that OA will modify the marine ecosystem, human communities will feel the effects of OA only when it alters economically and socially important marine ecosystem services (Cooley et al. 2009), but it will be too late at that time to introduce policies and actions to mitigate the greatest changes. There is no need to be alarmist, but there is an urgent need to develop strategies to provide decision-makers with tractable and sustainable solutions (Dupont and Thorndyke 2009). This interaction between biologists and economists will enhance the credibility of environmental research in the eyes of the public and decision-makers and will shed new light on the consequences of OA.

From the data presented here, it appears that despite the large amount of scientific data published over the last decade, a significant gap persists regarding their applicability by economists to assess the impacts of OA in policy-relevant terms. Therefore, there is an urgent need to develop research programmes involving close collaborations between biologists and economists in order to gain insights into “bio-socio-economic” impacts of OA. Even if it is currently risky to extrapolate from controlled short experiments to the natural environment, the complexity of ecosystem-level effects should not be an obstacle preventing the use of existing data by economists in their models. Rather, there needs to be a clear recognition of the uncertainty transferred between data and model types. Biologists and economists should adopt a common language, while at the same time accepting the rules and constraints of each field. As scientists, we have a duty to provide policy makers and public with reliable risk assessments, but it may be time to propose some solutions. Another challenge facing “bio-socio-economics” is the fact that OA cannot be disconnected from other global changes that are occurring such as climate change. Policy recommendations will have to tackle all of these effects simultaneously rather than focusing on OA alone. At the same time, policies addressing other types of global change should be considered with awareness of how they will impact acidification; for example, many apparent geoengineering “solutions” proposed for climate change mitigation will not help ameliorate ocean acidification (Williamson and Turley in press). The best policy choices will address multiple anthropogenically caused problems at the same time. Although we may never be able to predict all OA’s impacts exactly, we can manage the marine environment for maximum resilience, so it can better withstand all the pressures affecting it.

We hope the challenge posed by OA will stimulate multidisciplinary research and interactions between disciplines, and it will generate integrative models that will be widely useful in environmental management well beyond the problem of OA. As this work proceeds, “bio-socio-economic” impact assessment should be developed in an integrative way by teams with mixed expertise and not retained within a single discipline (Wam 2010), to achieve these goals.

## Electronic supplementary material

Below is the link to the electronic supplementary material.

Supplementary material 1 (DOCX 22 kb)
