# Supplementary material for: Towards improved socio-economic assessments of ocean acidification’s impacts
Source: Mar Biol. 2012 Aug 21;160(8):1773–87. doi: 10.1007/s00227-012-2031-5 (PMC3873077; doi:10.1007/s00227-012-2031-5)
Supplement: Supplementary file 1 — Supplementary material 1 (DOCX 22 kb) [file 227_2012_2031_MOESM1_ESM.docx]

**Supplementary Data:**

Ocean acidification may affect several ecosystems and their organisms that have biological and socio-economic importance.

**Table 1**

| **Ecosystems and organisms likely to be affected by ocean acidification** | **Biological importance** | **Socio-economic importance** | **Impacts of ocean acidification** |
| --- | --- | --- | --- |
| Coral reefs | - Major marine ecosystem - Central role in sustaining the biodiversity in the oceans realm (host 1/3 of all marine life) - Nursery for numerous organisms - Carbon export to the open sea - Corals as major habitat builders (ecosystem engineer) | - Coastal protection - Provide various resources, including drugs (antibacterial, antimitotic…) - Materials for construction - Food source for about 500 M people - Tourism industry, recreational and leisure - Entertainment (e.g. films and documentaries) - Aquarium market - Recreational and commercial fisheries | Weakens carbonate skeletons and reduces coral growth, narrows distribution range, exacerbates temperature effects, shifts in species composition, |
| Coralligen  (gorgonians, red coral, crustose coralline algae…) | - High biological diversity - Habitat - Spawning site - Inducer for larval invertebrate settlement (crustose coralline algae) | - Ecosystem maintenance - Recreational fisheries - Recreational diving - Entertainment (e.g. films and documentaries) - Jewelry (red coral) - Use for construction and as a soil conditioner (crustose coralline algae) | Very few data available, potential effect by reduction of calcification |
| Pelagic ecosystems | - Very large ecosystems (open ocean) - Key element in ocean productivity and food webs - Very large reservoir of marine organisms - Very important carbon reservoir - Nutrient cycling - Biogas production and flux to atmosphere | - Globally important source of oxygen - Globally important source of primary production supporting major food webs - Source of genes and drugs - Commercial fisheries - Recreational fisheries - Carbon sequestration | Shifts in species composition, potential effect on plankton growth and productivity |
| Plankton species  (foraminifera, coccolithophores, pteropods) | - Major component of the food web - Major component of marine ecosystems - Large primary production - Major elements in the carbon cycle - Calcifier species are major elements for long-term landscape builders (sedimentary rocks) | - Globally important source of oxygen for some species - Carbon sequestration - Food source for fishes - Biofuels - Genetic resources, drugs… | Likely important, potential effect by change of calcification and productivity |
| Macroalgae and seagrass meadows | - *Seagrass* beds: spawning site, nursering grouds, high biodiversity - Oxygen production - Habitat, high biodiversity - Food supply for large grazers - High productivity - Nutrient cycling | - Food source - Biofuels, alginates and land fertilizer - Oxygen production - Sediment stabilization - Commercially harvested species - Coastal protection (by reducing water currents) - Water purification - Recreational and commercial fisheries - Ecosystem maintenance - Recreational diving | CO_2_ entichment has fertilizing effect but alteration of species composition and biodiversity could occur.  Some species may benefit from OA (increase in productivity) |
| Estuarine environment | - Interface between land and sea leading to an highly variable environment - Important habitat for birds (particularly migratory birds) | - Nursery for commercial fish and invertebrates - Key transport hubs - Commercial, industrial, recreational fisheries - Aquaculture activities - Bird watching - Ecotourism - Most preferred residential locations and harbours | Vulnerable to multiple stressors including acidification from freshwater input, ocean acidification will be stronger in low salinity waters |
| Deep-sea | - Largest (but yet least-known) habitat on earth - Largest store of carbon (apart from rocks) on planet - High biodiversity - Important endemic / specific organisms - Cold-water corals as ecosystem engineer and support of deep-water ecosystems - Important part of food web - Important in remineralization of organic matter and nutrient recycling, important carbon sink role | - Habits for deep-sea fishes (nursery) - Source of new genes, enzymes, chemicals and drugs - Commercial fisheries - Entertainment (e.g. films and documentaries) | Weakens carbonate skeletons and reduces coral growth, shifts in species composition, change in nutrient cycles |
| Polar regions | - Original ecosystems with highly specific (endemic) organisms - Significant carbon cycling role (Southern Ocean) | - Source of new genes and drugs - Tourism, touristic cruise - Commercial fisheries - Entertainment (e.g. films and documentaries) - Home to charismatic species such as polar bears | Ocean acidification will be stronger in cold waters, but only few data on sensitivity of polar organisms |
| Mollusks (oysters, musels, scallops, nudibranchs, sea snails, limpets, squids…) | - Second largest [marine](http://en.wikipedia.org/wiki/Marine_biology) phylum (about 17% of all the known marine [organisms](http://en.wikipedia.org/wiki/Organism)) - Important part of food web - Landscape builders | - Important human food source (sea foods: oysters, mussels, scallops…) - Aquaculture - Jewelry (pearls, mother-of-pearl) - Source of drugs (antibacterial, antifungal, anti-inflammatory, antimitotic…) - Genetic resources - Source of textile fibers - Aquariology | Weakens carbonate shells and reducesreproduction, growth, shifts in species composition |
| Echinoderms (Sea urchins, sea cucumbers, starfish…) | - Important part of food web - Keystone species and ecosystem engineers | - Human food source (sea urchins, sea cucumbers - Genetic resources - Major animal model for developmental biology and regenerative medicine (sea urchins, starfish) | Weakens carbonate skeletons and reduces growth, possible total extinction of some species (brittlestars) |
| Sponges | - Ecosystem engineer, landscape formation, habitat for important fish species - Water filtration | - Sponge culture - Important source of drugs - Recreational diving - Genetic resources, enzymes antibacterial compounds | Weakens carbonate skeletons and reduces growth |
| Crustaceans  (crabs, prawns, lobsters, crayfish…) | - First largest animal phylum - Important part of food web | - Important human food source - Use in aquaria - Source of drugs and chitin | Inhibition to stimulation of growth according to species, shifts in species composition |
| Marine mammals (whales, dolphins, seals…) | - Plankton-eaters and source of carbon for deep-sea ecosystems (carcass) - Top predators and food source - Role in nutrient cycle (e.g. Southern Ocean | - Cultural role (charismatic species) - Ecotourism such as whale watching - Fishes by some cultures - Entertainment (e.g. films and documentaries) | Possible indirect effects by change in the food web |
| Fishes  (herrings, sardines, anchovies, tunas, cods, flounders, sharks…) | - Important part of food web - Forage species for top predators - Some are top predators | - Commercial fisheries - Aquaculture - Human food (almost 80% of the world catch) - Oil production - Fish meal provision - Recreational fisheries - Cultural role (charismatic species and cultural heritage) - Entertainment (e.g. stories, films and documentaries) | Effects uncertain, disturbances reported in development, behaviours and predation abilities |
| Bacterial community | - Drivers of major biogeochemical cycles (decomposition of organic matter, nutrient regeneration…) - Most production is cycled through the microbial loop - Some are major actor in O_2_ production and marine productivity - Food source for small heterotrophic zooplankton and benthic feeders | - Ecosystem maintenance - Nutrient cycling - Sewage treatment - Source of new genes and drugs | Potential alteration of microbial processes, potential impact on biogeochemical cycles and climate regulation |
| Jellyfish | - Important part of food web - Biological model for medical research | - Commercial fisheries - Competitors with fishermen - Obstacle for beach activities, no swimmers - Genetic resources - Source of drugs and medical compounds | Few contrasting data available, possible indirect effects |
